# Remarks on Different Subjects

**Published:** 1867-02

**Authors:** N. Holton

**Affiliations:** Buda, Ill.


### ARTICLE XIII.

### REMARKS ON DIFFERENT SUBJECTS.

By N. HOLTON, M.D., Buda, Ill.

Read to the Members of the Military Tract Medical Association, Dec. 11, 1866.

After amputation by what is known as the flap operation, there is a redundancy of muscle in many cases. This I have often seen, both in civil and military surgery, and, because of this happening to me, I thought it might or had occurred in the experience of others. It does not succeed well if the integuments are drawn too tightly to cover the muscles in coaptating them, and, on this account, it becomes necessary to reämputate the muscles before the integuments can be properly adapted together so as to secure union by the first intention.

It occurred to me that, in most cases, by grasping the parts from which the flap was to be made, and retracting the skin, the difficulty above spoken of might be avoided.

In some cases of amputations by the flap method, the weight of the flaps has been thought to be an objection. This objection, perhaps, would not have as much weight in civil as military practice, but some in both.

In a case where I amputated below the knee, some weeks ago, the posterior flap being formed of the muscles of the calf of the leg, and this, being the principal flap, was quite heavy. If this flap is allowed to draw down its whole weight, it will be quite sure to destroy the vitality of the tissues where they are drawn across the tibia, and, consequently, result in sloughing. This was likely to be the result in the case above alluded to, but upon being carefully supported by a double inclined plane curved splint, the patient went on well again. They may be supported by cushions or pillows sufficiently for all practical purposes. In cases where there is considerable suppuration or erysipelatous tendency, I am in the habit of prescribing the muriated tincture of iron freely, and have never had patients affected with traumatic erysipelas or hospital gangrene where it was used as a prophylactic. I am indebted to Prof. E. ANDREWS, of the Chicago Medical College, for this idea.

*Two Cases of Spina Bifida.*

On June 29th, 1866, I was called to attend Mrs. M. in her first labor, and, after a little more than usually hard time, she was delivered of a large male child, affected with spina bifida. The unusually hard labor was caused, I thought, by the cord being around the body just above the pelvis. Instead of a tumor on the back, the tissues were gone considerably below the surrounding parts in the region of the two first lumbar vertebræ, and the posterior half of the vertebræ seemed to be gone. The parietal and frontal bones were less ossified than usual, leaving the parietal bones widely separated along the median line and extending into the frontal bone as far as a level of the superciliary ridge. Otherwise, the child was well developed. I thought that if the parts could be sufficiently supported, that lacked development, or had lost their integrity, that the function of organization might go on and the cranial bones become ossified. To carry out this, I ordered compress and bandage, and, under this management, the ulcer on the spine became covered with a thin, parchment-like membrane, but, perhaps for want of thoroughness on the part of the parents, or from some other cause, the tumor grew and the contents of the cranium protruded more and more beyond the bones. About the 10th of October, there came a hole in the tumor of a very minute size, and on the 14th the child died.

There was nothing uncommon about the other case, and it did not live but a few weeks. It was accompanied by talipes, a very usual thing, I believe, in spina bifida. The mother of the first case was severely frightened by a horse kicking while she was in the buggy, three months before her accouchement, and the second fell on the ice in the early part of gestation, hurting her back severely. Whether these, or the fact of the cord being tightly drawn around the body of one child, had anything to do with these cases or not, I am not satisfied.

Prof. D. Brainard, of Rush Medical College, in the catalogus for 1847 and 1848, says, "Cure of Spina Bifida by Injections of Iodine." But, unfortunately for the practice, Dr. Brainard's case soon after died; and as that was so long ago, and the practice not yet confirmed, I suppose it is a failure. Can these cases be cured? I confess I do not know.

In conversation with Dr. H. Nance, of Kewanee, upon spina bifida, he said there was a case in his town, in the last cervical or first dorsal vertebra, of small size, in which the tumor was covered with a strong membrane; and that the child was doing well, could walk, and do anything that other children do.

*A Case of Chronic Inflammation of the Urinary Organs.*

This, I wish to include the kidneys, ureter, bladder, and urethra.

On the 11th of September, 1866, I was consulted by Mr. H., for what he called gravel, and, on inquiry, I elicited the following history:—He had been in the cattle business, in Missouri and Texas, through the season, almost constantly riding on horseback, and had noticed a diminishing stream of urine, till, suddenly, he was unable to pass it at all, (this was two weeks before I saw him,) and compelled him to consult a physician, who relieved him with the catheter. When I first saw him, he could not pass urine, and he was again relieved by the use of the catheter, without difficulty; but the urine was largely mixed with blood, and some bloody mucus was entangled in the eyes of the catheter. At this time, there was very slight constitutional disturbance, and, after a careful examination, could find but little soreness or other enidence of organic disease. He had a good appetite, and felt pretty well generally. From these data, I gave the patient encouragement that he would recover his health in a short time.

He went on well until the 27th of the same month, when, suddenly, he was taken worse than ever—urine retained; some throbbing pain in region of prostate gland, and pain in the back; and a slight febrile movement generally, together with great nervousness.

These symptoms were relieved, and recurred again and again until the 20th of October, when I told him the full extent of the mischief, according to my view of the case, and that he could not be cured for at least three months, and, perhaps, a longer time. This was such poor consolation to him that he concluded to dispense with my services and seek aid elsewhere.

On the 11th of October, I was again summoned to see Mr. H. Found him unable to pass urine; laborious breathing; a black, dry tongue; pulse very frequent, small, and weak; entire loss of appetite; and unable to raise up in bed. At this time, I told him I would not take charge of the case unless he would allow counsel. This was readily assented to, and I sent for E. ANDREWS, M.D., of Chicago, who promptly responded, verified the diagnosis, and we agreed upon a course of treatment; but he gradually run down, and died on the 11th of November.

The reason I report this case is, that such cases are very infrequent in my practice, and the absence or obscurity of prominent symptoms, leading one to fully appreciate the importance of the case he has to deal with at first; the great difficulty in curing them when they are known; and the great lack of authority on the subject, within the reach of the country pr ctitioner. The treatment, I understand to be astringent injections into the bladder, the best of which is nit. silver, in weak solution at first, and increased in strength as it can be tolerated; and the alkalies, buchu, pareira brava, tr. ferri chloridi, potassa brom., and other remedies of this class, by the mouth; rest, and care that the bowels are kept in a soluble condition.

May I express the wish, that some member of the profession, of large experience in this class of diseases, will write a monograph upon them.

